# Association between prognostic nutritional index and survival of patients with oral cancer: a meta-analysis

**DOI:** 10.3389/fonc.2025.1698656

**Published:** 2025-11-19

**Authors:** Guodong Jia, Zhen Wang

**Affiliations:** 1 Department of General Dentistry, Shanghai Ninth People’s Hospital, Shanghai Jiao Tong University School of Medicine, Shanghai, China; 2 College of Stomatology, Shanghai Jiao Tong University, National Center for stomatology, Shanghai, China; 3 National Clinical Research Center for Oral Diseases, Shanghai Key Laboratory of Stomatology, Shanghai Research Institute of Stomatology, Shanghai, China; 4 Department of Oral and Maxillofacial-Head and Neck Oncology, Shanghai Ninth People’s Hospital, College of Stomatology, Shanghai Jiaotong University School of Medicine, Shanghai, China; 5 National Clinical Research Center for Oral Diseases, Shanghai Key Laboratory of Stomatology & Shanghai Research Institute of Stomatology, Shanghai, China

**Keywords:** oral cancer, oral squamous cell carcinoma, prognostic nutritional index, progression, survival

## Abstract

Oral cancer (OC) remains a major global health burden with unsatisfactory survival outcomes. The prognostic nutritional index (PNI), derived from serum albumin and lymphocyte counts, has been proposed as a simple biomarker of nutritional and immune status. This meta-analysis was performed to summarize the association between PNI and survival of patients with OC. We systematically searched PubMed, Embase, and Web of Science for longitudinal observational studies which compared low versus high PNI groups and reported hazard ratios (HRs) with 95% confidence intervals (CIs) for overall survival (OS) and/or progression-free survival (PFS). Pooled analyses were performed using random-effects models accounting for heterogeneity. A total of 16 cohorts were included. Low PNI was significantly associated with poorer OS (HR: 2.68, 95% CI: 2.00 to 3.58; I² = 64%) and PFS (HR: 1.99, 95% CI: 1.66 to 2.39; I² = 0%). Subgroup analyses revealed a stronger association in patients with mean age ≥ 65 years for both OS (HR: 3.49 vs. 1.48; *p* for subgroup difference = 0.001) and PFS (HR: 2.76 vs. 1.42; *p* = 0.002) as compared to those < 65 years. Meta-regression confirmed age as a significant source of heterogeneity (OS coefficient = 0.060, *p* = 0.004; adjusted R² = 81.4%; PFS coefficient = 0.055, *p* = 0.02; adjusted R² = 100.0%). Low PNI before treatment is associated with adverse survival in patients with OC, particularly in older populations. These findings support the integration of PNI into prognostic assessment in OC management.

**Systematic review registration:**

PROSPERO, identifier CRD420251139755.

## Introduction

Oral cancer (OC) is a major global health problem, accounting for a substantial proportion of head and neck malignancies and contributing significantly to cancer-related morbidity and mortality worldwide (1, 2). The vast majority of OC cases are histologically diagnosed as oral squamous cell carcinoma (OSCC), representing over 90% of all oral malignancies (3). Despite advances in multimodal treatment strategies—including surgery, radiotherapy, chemotherapy, and more recently, immunotherapy—the prognosis of OC remains unsatisfactory, particularly for patients diagnosed at advanced stages (4, 5). Five-year survival rates have shown only modest improvement over the past decades, largely due to high rates of recurrence and metastasis (6). As a result, there is an urgent need to identify reliable and accessible prognostic predictors that can complement traditional clinicopathological factors, thereby improving risk stratification and guiding personalized treatment strategies in OC.

The prognostic nutritional index (PNI), calculated as serum albumin (g/L) + 5 * lymphocyte count (10^9^/L), is a simple and objective biomarker that reflects both the nutritional and immune status of cancer patients, which was firstly proposed by Onodera et al. in 1984 (7, 8). Hypoalbuminemia and lymphopenia may predispose patients to impaired immune surveillance, systemic inflammation, and poor treatment tolerance, ultimately leading to adverse clinical outcomes (9, 10). These mechanisms suggest that a low PNI could contribute to tumor progression and worse prognosis in OC. Although accumulating observational studies have investigated the prognostic value of PNI in OC, their findings have been inconsistent, with some demonstrating strong associations with survival (11–23) while others report null or weaker effects (24, 25). Variability in study design, sample size, cutoff determination, and patient characteristics may explain these discrepancies. A prior meta-analysis published in 2023 summarized evidence from 10 studies (26), but new data have since emerged. Therefore, an updated meta-analysis incorporating recent studies and exploring age-related effects was warranted to provide a more comprehensive and current understanding of the prognostic value of PNI in OC. To address this uncertainty, we conducted a comprehensive meta-analysis to quantitatively evaluate the association between PNI and survival outcomes, including overall survival (OS) and progression-free survival (PFS), in patients with OC. This study aims to clarify the prognostic significance of PNI and explore potential sources of heterogeneity, thereby providing stronger evidence for its clinical utility in the prognostic assessment of OC.

## Methods

The study was conducted in accordance with the PRISMA 2020 guidelines (27) and the Cochrane Handbook (28) for Systematic Reviews of Interventions, ensuring methodological rigor in study selection, data extraction, statistical analysis, and result interpretation. The protocol was prospectively registered with PROSPERO (ID: CRD420251139755).

### Literature search

A comprehensive literature search was performed in PubMed, Embase, and Web of Science, utilizing a broad set of search terms that integrated the following keywords and concepts (1): “prognostic nutritional index” OR “prognostic nutrition index” OR “PNI” (2); “oral squamous cell carcinoma” OR “oral cancer” OR “oral cavity cancer” OR “mouth neoplasm” OR “oral-pharyngeal cancer”; and (3) “mortality” OR “prognosis” OR “survival” OR “death” OR “recurrence” OR “progression”. The search was limited to human studies and included only full-text articles published in English in peer-reviewed journals. To ensure completeness, we also manually screened the reference lists of relevant original and review articles for additional eligible studies. The search covered all publications from database inception up to June 8, 2025. The detailed search strategy for each database is shown in **Supplementary File 1**.

### Study eligible criteria

We applied the PICOS framework to define the inclusion criteria:

Population (P): Patients with histologically confirmed OC, regardless of stage or treatment status.

Intervention/Exposure (I): PNI categorized as low vs. high using study-defined cutoff values. Patients with a low PNI was considered as exposure, with the cutoff values for defining a low PNI consistent with the methods used in the original studies.

Comparator (C): Patients with high PNI.

Outcomes (O): Primary Outcome: Overall survival (OS); Secondary Outcome: Progression-free survival (PFS). OS was generally defined as the time from treatment initiation to death from any cause, while PFS referred to the time from treatment initiation to either disease progression or death, whichever occurred first. PFS, disease-free survival, and recurrence-free survival were combined under the unified term “PFS”, as these outcomes share a consistent definition representing the time from treatment initiation to disease progression, recurrence, or death.

Study design (S): Longitudinal follow-up studies (prospective or retrospective cohorts, nested case-control studies, and *post-hoc* analysis of randomized controlled trials) reporting hazard ratios (HRs) and 95% confidence intervals (CIs) for the association between PNI (low vs. high) and survival outcomes.

Case reports, case series, reviews, editorials, meta-analyses, animal studies, studies not limited to patients with OC, lacking PNI as exposure, or lacking survival outcomes or insufficient data to calculate HRs with 95% CIs were excluded. For duplicate reports from the same patient cohort, only the study with the largest sample size was included.

### Study quality evaluation

Two reviewers independently conducted the literature search, screened studies, assessed methodological quality, and extracted data. Any discrepancies were resolved through discussion and consensus between the two authors. The quality of included studies was evaluated using the Newcastle–Ottawa Scale (NOS) (29), which examines study selection, control of confounding variables, and outcome assessment. The NOS assigns scores ranging from 1 to 9, with a score of 8 or above indicating high methodological quality. Potential selection bias is captured under the “Representativeness of the exposed cohort” domain, which awards a point for consecutive, random, or prospective inclusion of participants. Confounding control is represented by the domains “Control for age and sex” and “Control for other confounding factors,” which reflect adjustment for key prognostic variables in multivariate analyses. The NOS was chosen for its widespread acceptance and methodological compatibility with previous meta-analyses in this field.

### Data collection

The data collected for the meta-analysis included study details (author, year, country, and design), patient characteristics (diagnosis, number of patients in each study, mean age, sex distribution, cancer stage, and main treatment), exposure details (timing for evaluation PNI, methods for determining the cutoff of PNI, and cutoff values for defining a low PNI), median follow-up durations, outcomes reported, and covariates adjusted for in the regression models evaluating the association between a low PNI and survival outcome of patients with OC.

### Statistical analysis

We used HRs and 95% CIs to assess the association between PNI and survival outcomes of patients with OC. HRs and their standard errors were either directly extracted or derived from reported 95% confidence intervals or p-values, followed by logarithmic transformation to stabilize variance and achieve a normal distribution (28). If multiple HRs were reported from different models, we used the one with the most complete adjustment. Heterogeneity was assessed using the Cochrane Q test and the I² statistic (30), with a *p*-value < 0.10 indicating significant heterogeneity and I² values of < 25%, 25–75%, and > 75% indicating low, moderate, and high heterogeneity, respectively. A random-effects model was applied to synthesize the data, allowing for variability across studies (28). To assess the stability of the results, sensitivity analyses were conducted by sequentially excluding each study. For outcomes involving at least ten datasets, predefined subgroup analyses were conducted based on mean ages of the patients (< 65 vs. ≥ 65 years), proportions of men, cancer stage (stage I-IV vs. advanced stage, such as stage III-IV and recurrent OC), cutoff values of PNI, follow-up durations, analytic models (univariate vs. multivariate analyses), and study quality scores. In general, subgroup analyses were stratified using the median values of continuous variables to ensure balanced groupings. In addition, the univariate meta-regression analysis was performed to evaluate the influence of study characteristics on the association between PNI and survival outcomes, including mean patient ages, proportions of men, cutoff values of PNI, follow-up durations, and NOS. Publication bias was evaluated through funnel plot visualization and assessed for asymmetry using Egger’s regression test (31). All analyses were performed using RevMan (Version 5.3; Cochrane Collaboration, Oxford, UK) and Stata (Version 17.0; Stata Corporation, College Station, TX, USA).

## Results

### Study inclusion

**Figure 1:**
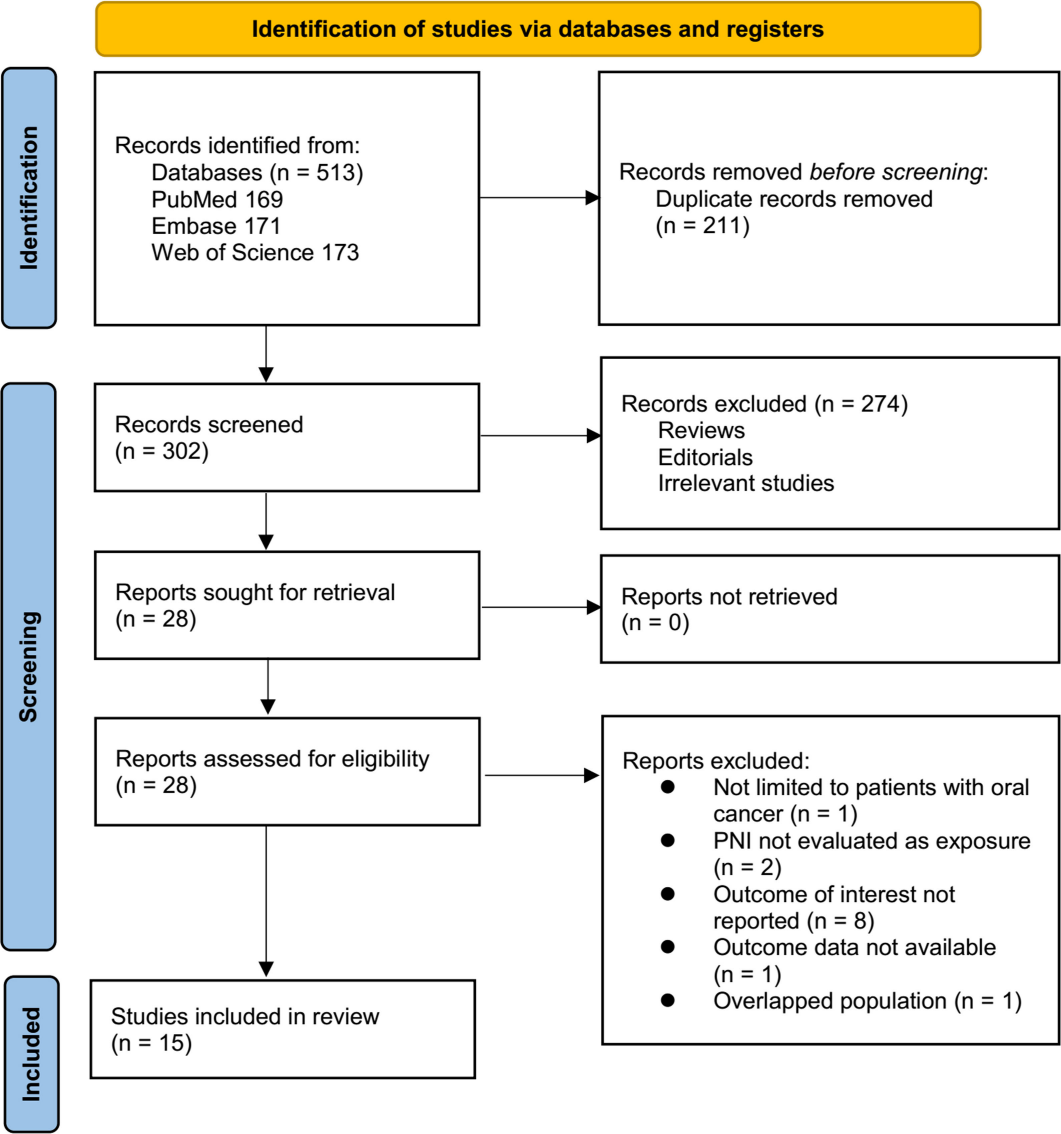
Flowchart of database search and study inclusion.

The study selection process is shown in **Figure 1**. We first identified 513 records from the three databases. Following the removal of 211 duplicate records, 302 articles underwent title and abstract screening. Of these, 274 were excluded for not aligning with the objectives of the meta-analysis. The remaining 28 full-text articles were assessed independently by two reviewers, resulting in the exclusion of 13 studies for specific reasons detailed in **Figure 1**. At last, 15 studies were included in the subsequent analysis (11–25).

### Summary of study characteristics

The main features of the 16 cohorts from 15 studies included in this meta-analysis are summarized in **Table 1**. These cohorts collectively enrolled 3,520 patients with OC, with one study (12) contributing two independent cohorts analyzed separately. The studies were conducted in China, Japan, and Taiwan (China) and were published between 2020 and 2025. Most cohorts (12–25) employed retrospective cohort designs, and one study was a prospective cohort study (11). The mean age of participants ranged from 54.0 to 79.0 years, and the proportion of male participants ranged from 45.9% to 90.3%. Cancer stage at diagnosis spanned I to IV, with three cohorts focusing specifically on advanced, recurrent, or metastatic disease (19, 20, 23). Treatment modalities varied and included surgery alone, surgery with adjuvant radiotherapy and/or chemotherapy, definitive chemoradiotherapy, and immune checkpoint inhibitors for recurrent/metastatic disease. PNI was assessed preoperatively or pretreatment in all cohorts, with cutoff values determined primarily by ROC curve analysis (13–16, 19–24), X-tile software (12), or previous study defined cutoff values (17, 18), while two cohorts used median values (11, 25). Follow-up durations ranged from 1.3 to 8.3 years. All 16 cohorts reported OS (11–25), while 12 also reported PFS (12–17, 19, 20, 22, 23, 25). Twelve cohorts adjusted for key confounders such as age, sex, tumor stage, and pathological factors etc. to a varying degree (11–17, 21–24), whereas four cohorts reported unadjusted analyses only (18–20, 25).

**Table 1:** Characteristics of the included studies.

Study	Country	Study design	Diagnosis	No. of patients	Mean age (years)	Men (%)	Cancer stage	Main treatment	Timing of PNI measuring	Methods for determining the cutoff of PNI	Cutoff value of PNI	Median follow-up duration (years)	Outcome reported	Variables adjusted
Bao 2020 (11)	China	PC	Primary oral cancer	1395	57.2	62.9	I-IV	Surgery (88.6%), Adjuvant therapy: None (48.0%), RT (14.6%), CT (14.9%), CRT (22.5%)	Preoperative	Median	49.3	8.3	OS	Age, sex, occupation, education level, residence, clinical classification (TNM stage), pathological grading, surgery therapy, adjuvant therapy, and recruitment time
Wu 2020 C1 (12)	China	RC	OSCC	166	NR	53.6	I-IV	Curative tumor resection. Postoperative radiotherapy if node-positive	Preoperative	X-tile software derived	47.4	4.0	OS and PFS	Age, sex, tumor size, pathological grade, cervical node metastasis, and clinical stage
Wu 2020 C2 (12)	China	RC	OSCC	167	NR	51.5	I-IV	Curative tumor resection. Postoperative radiotherapy if node-positive	Preoperative	X-tile software derived	47.4	6.0	OS and PFS	Age, sex, tumor size, pathological grade, cervical node metastasis, and clinical stage
Yoshida 2020 (13)	Japan	RC	Advanced OSCC	47	79.0	48.9	III-IV	Definitive chemoradiotherapy	Pre-treatment	ROC curve analysis	42.7	2.9	OS and PFS	Age, sex, tumor stage, WPOI, primary site, and RECIST
Abe 2021 (14)	Japan	RC	OSCC	102	65.6	71.6	I-IV	Radical surgical therapy	Preoperative	ROC curve analysis	42.9	4.0	OS and PFS	Age, sex, CRP, tumor grade, stage, lymphovascular invasion, vascular invasion, perineural invasion, close margin, and postoperative treatment
Watabe 2021 (15)	Japan	RC	OSCC	110	68.0	55.5	I-IV	Radical surgery with curative intent	Preoperative	ROC curve analysis	52.4	5.7	OS and PFS	Age, sex, tumor stage, histological grade, lymphatic invasion, vascular invasion, extracapsular spread, surgical margins, NLR, PLR, and LMR
Yamagata 2022 (18)	Japan	RC	OSCC	155	70.4	61.3	I-IV	Surgery only (56.8%), Surgery + RT ± CT (27.8%), RT only (15.5%)	Pre-treatment	Previous study determined	49.3	4.0	OS	None
Kubota 2022 (17)	Japan	RC	OSCC	183	66.0	56.3	I-IV	Surgery only (64.5%), Surgery + RT/CRT (16.9%), SSIACRT (18.6%)	Pre-treatment	Previous study determined	52.4	6.3	OS and PFS	Age, sex, tumor stage, and treatments
Yoshimura 2022 (19)	Japan	RC	OSCC	112	68.0	61.6	I-IV	Surgery with or without chemoradiotherapy	Preoperative	ROC curve analysis	50.6	3.7	OS and PFS	None
Fang 2021	Taiwan (China)	RC	OSCC	360	59.0	90.3	I-IV	Primary curative surgery + neck dissection, with or without chemoradiotherapy	Preoperative	ROC curve analysis	51.8	3.9	OS and PFS	Age, sex, PNE, ENE, cell differentiation, adjuvant CCRT, surgical margins, CCI, NLR, and PLR
Li 2024 (21)	China	RC	OSCC	262	NR	66.8	I-IV	Radical surgery with or without chemoradiotherapy	Preoperative	ROC curve analysis	45.5	4.2	OS	Age, sex, and TNM stage
Kikuta 2024 (20)	Japan	RC	Recurrent or metastatic oral cancer	31	68.6	74.2	Recurrent or stage IV	ICIs, either as monotherapy (64.5%) or combined with chemotherapy (35.5%)	Pre-treatment	ROC curve analysis	40.7	3.3	OS and PFS	None
Song 2024 (25)	China	RC	OSCC	116	54.0	75.0	I-IV	Surgical resection of primary tumor and metastatic lymph nodes	Preoperative	Median	49.3	1.3	OS and PFS	None
Ohyama 2024 (24)	Japan	RC	OSCC	146	69.9	45.9	I-IV	Radical surgery with or without chemoradiotherapy	Preoperative	ROC curve analysis	51.4	2.5	OS	Age, sex, CT-sarcopenia, and LMR
Ooyama 2025 (23)	Japan	RC	Recurrent or metastatic oral cancer	42	75.5	52.4	Recurrent or stage IV	ICIs	Pre-treatment	ROC curve analysis	36.4	2.5	OS and PFS	Age, sex, ECOG-PS, RECIST, differentiation, WPOI, and CPS
Fukuzawa 2025 (22)	Japan	RC	Tongue SCC	126	67.0	54.8	I-IV	Radical surgery with or without chemoradiotherapy	Preoperative	ROC curve analysis	51.1	4.9	OS and PFS	Age, sex, BMI, pathological stage, and tumor grade

BMI, body mass index; CCI, Charlson comorbidity index; CCRT, concurrent chemoradiotherapy; CPS, combined positive score; CRP, C-reactive protein; CRT, chemoradiotherapy; ECOG-PS, Eastern Cooperative Oncology Group performance status; ENE, extranodal extension; ICIs, immune checkpoint inhibitors; LMR, lymphocyte-to-monocyte ratio; NLR, neutrophil-to-lymphocyte ratio; NR, not reported; OSCC, oral squamous cell carcinoma; PLR, platelet-to-lymphocyte ratio; PNE, perineural extension; RECIST, Response Evaluation Criteria in Solid Tumors; RT, radiotherapy; SCC, squamous cell carcinoma; SSIACRT, superselective intra-arterial chemoradiotherapy; TNM, tumor-node-metastasis; WPOI, worst pattern of invasion.

### Study quality evaluation

Study quality was assessed using the NOS, with total scores ranging from 6 to 9, indicating moderate to high methodological quality (**Table 2**). Eleven cohorts scored ≥ 8, reflecting good quality with representative populations, well-defined exposure and outcome assessments, and adequate follow-up durations (11–17, 21, 22, 24). The remaining five cohorts scored 6 or 7, primarily due to limited adjustment for confounders or incomplete follow-up information (18–20, 23, 25). Overall, the included studies demonstrated acceptable methodological quality, supporting the reliability of the pooled findings.

**Table 2:** Study quality evaluation via the Newcastle-Ottawa scale.

Study	Representativeness of the exposed cohort	Selection of the non-exposed cohort	Ascertainment of exposure	Outcome not present at baseline	Control for age and sex	Control for other confounding factors	Assessment of outcome	Enough long follow-up duration	Adequacy of follow-up of cohorts	Total
Bao 2020 (11)	1	1	1	1	1	1	1	1	1	9
Wu 2020 C1 (12)	1	1	1	1	1	1	1	1	1	9
Wu 2020 C2 (12)	1	1	1	1	1	1	1	1	1	9
Yoshida 2020 (13)	1	1	1	1	1	1	1	0	1	8
Abe 2021 (14)	1	1	1	1	1	1	1	1	1	9
Watabe 2021 (15)	1	1	1	1	1	1	1	1	1	9
Yamagata 2022 (18)	1	1	1	1	1	0	0	1	1	7
Kubota 2022 (17)	1	1	1	1	1	1	1	1	1	9
Yoshimura 2022 (19)	1	1	1	1	1	0	0	1	1	7
Fang 2021	1	1	1	1	1	1	1	1	1	9
Li 2024 (21)	1	1	1	1	1	1	1	1	1	9
Kikuta 2024 (20)	0	1	1	1	0	0	1	1	1	6
Song 2024 (25)	1	1	1	1	0	0	1	0	1	6
Ohyama 2024 (24)	1	1	1	1	1	1	1	0	1	8
Ooyama 2025 (23)	0	1	1	1	1	1	1	0	1	7
Fukuzawa 2025 (22)	1	1	1	1	1	1	1	1	1	9

### Association between PNI and OS in patients with OC

**Figure 2:**
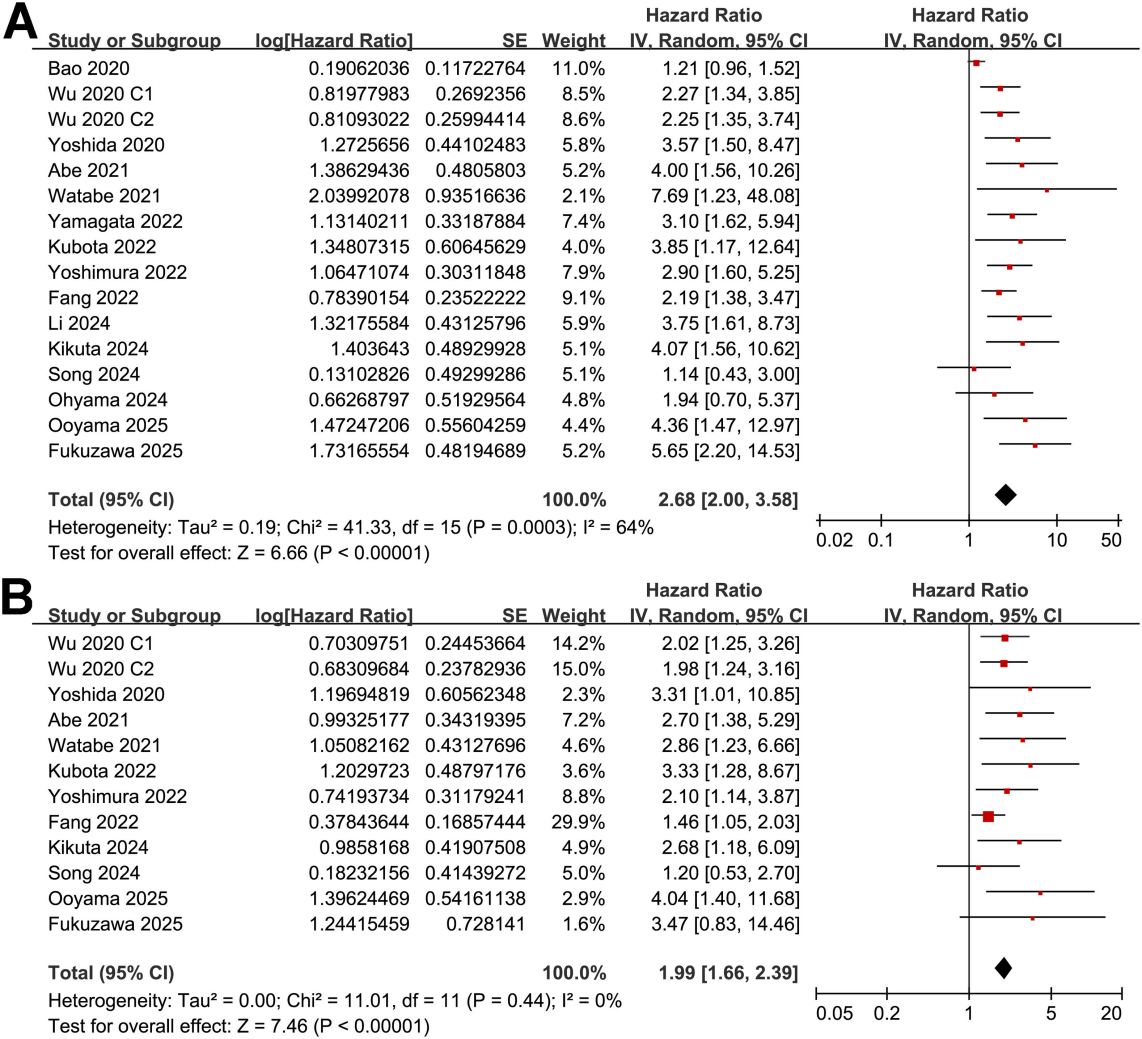
Forest plots for the meta-analysis of the association between a low PNI and survival outcome of patients with OC; **(A)** forest plots for the meta-analysis of the association between a low PNI and OS; and **(B)** forest plots for the meta-analysis of the association between a low PNI and PFS;.

A total of 16 cohorts (11–25) reported the association between PNI and OS in patients with OC. Significant heterogeneity was observed (*p* for the Cochrane Q test < 0.001; I^2^ = 64%). Pooled results from a random-effects model showed that, overall, a low PNI at baseline was associated with poorer OS in patients with OC (HR: 2.68, 95% CI: 2.00 to 3.58, *p* < 0.001; **Figure 2A**). Sensitivity analyses were performed by removing one dataset at a time, and the results remained stable (HR: 2.55 to 2.81, *p* < 0.05 for all comparisons). Further subgroup analysis showed a stronger association between a low PNI and OS in OC patients with mean ages ≥ 65 years as compared to < 65 years (HR: 3.49 vs. 1.48, *p* for subgroup difference = 0.001; **Table 3**). The association between a low PNI and OS was not significantly affected by the proportion of men, cancer stage, cutoff of PNI, follow-up duration, analytic models, or NOS (*p* for subgroup difference all > 0.05; **Table 3**). Of note, subgroup analyses markedly reduced heterogeneity in several strata—for example, in cohorts with mean age ≥ 65 years (I² = 0%), high-quality studies (I² = 14%), and shorter follow-up durations < 4 years (I² = 0%). Consistently, the results of univariate meta-regression analysis also showed that the mean ages of the patients were positively correlated with the association between a low PNI and worse OS in patients with OC (coefficient = 0.060, *p* = 0.004; **Table 4**), which substantially explained the source of heterogeneity (adjsuted R^2^ = 81.4%). Other variables including sex distribution, cutoff of PNI, follow-up duration, or study quality scores did not significantly correlate with the association between PNI and OS (*p* all > 0.05; **Table 4**).

**Table 3:** Results of subgroup analyses.

	OS					PFS				
Variables	No. of studies	HR (95% CI)	I^2^	*p* for subgroup effects	*p* for subgroup difference	No. of studies	HR (95% CI)	I^2^	*p* for subgroup effects	*p* for subgroup difference
Mean age (years)										
< 65 years	3	1.48 [0.95, 2.31]	62%	0.08		2	1.42 [1.05, 1.93]	0%	0.02	
≥ 65 years	10	3.49 [2.64, 4.63]	0%	< 0.001	0.001	8	2.76 [2.04, 3.74]	0%	< 0.001	0.002
Men (%)										
< 60%	8	2.81 [2.13, 3.72]	0%	< 0.001		7	2.37 [1.81, 3.11]	0%	< 0.001	
≥ 60%	8	2.40 [1.58, 3.63]	73%	< 0.001	0.53	5	1.80 [1.35, 2.41]	19%	< 0.001	0.17
Cancer stage										
I-IV	13	2.49 [1.82, 3.40]	65%	< 0.001		9	1.89 [1.56, 2.29]	0%	< 0.001	
Advanced only (III-IV or recurrent)	3	3.93 [2.26, 6.83]	0%	< 0.001	0.16	3	3.17 [1.79, 5.60]	0%	< 0.001	0.09
Cutoff of PNI										
< 49	7	2.87 [2.18, 3.78]	0%	< 0.001		7	2.17 [1.69, 2.79]	0%	< 0.001	
≥ 49	9	2.40 [1.58, 3.64]	71%	< 0.001	0.48	5	2.01 [1.42, 2.86]	25%	< 0.001	0.73
Follow-up duration (years)										
< 4	7	2.54 [1.92, 3.35]	0%	< 0.001		6	1.91 [1.37, 2.66]	27%	< 0.001	
≥ 4	9	2.83 [1.83, 4.37]	74%	< 0.001	0.68	6	2.30 [1.76, 3.00]	0%	< 0.001	0.39
Analytic models										
Univariate	4	2.68 [1.73, 4.14]	25%	< 0.001		3	1.93 [1.27, 2.93]	0%	0.002	
Multivariate	12	2.72 [1.92, 3.86]	67%	< 0.001	0.96	9	2.08 [1.67, 2.61]	11%	< 0.001	0.75
NOS										
6~7	5	2.85 [1.94, 4.18]	14%	< 0.001		4	2.15 [1.39, 3.32]	17%	< 0.001	
8~9	11	2.63 [1.84, 3.77]	68%	< 0.001	0.77	8	1.98 [1.60, 2.44]	3%	< 0.001	0.74

CI, confidence interval; HR, hazard ratio; I², inconsistency index; NOS, Newcastle-Ottawa Scale; OS, overall survival; PFS, progression-free survival; PNI, prognostic nutritional index;

**Table 4:** Results of univariate meta-regression analysis.

Variables	HR for OS				HR for PFS			
	Coefficient	95% CI	P values	Adjusted R^2^	Coefficient	95% CI	P values	Adjusted R^2^
Mean age (years)	0.060	0.024 to 0.095	0.004	81.4%	0.055	0.013 to 0.098	0.02	100%
Men (%)	-0.0068	-0.0310 to 0.0173	0.55	0%	-0.012	-0.029 to 0.005	0.11	12.8%
Cutoff of PNI	-0.040	-0.110 to 0.030	0.24	11.4%	-0.046	-0.108 to 0.016	0.13	11.2%
Follow-up duration (years)	-0.12	-0.26 to 0.03	0.08	62.3%	0.070	-0.124 to 0.261	0.44	0%
NOS	-0.028	-0.296 to 0.241	0.83	0%	-0.0055	-0.2285 to 0.2175	0.96	0%

CI, confidence interval; HR, hazard ratio; NOS, Newcastle-Ottawa Scale; OS, overall survival; PFS, progression-free survival; PNI, prognostic nutritional index;

### Association between PNI and PFS in patients with OC

Further meta-analysis of 12 cohorts (12–17, 19, 20, 22, 23, 25) showed that a low PNI was also significantly associated with poorer PFS in patients with OC (HR: 1.99, 95% CI: 1.66 to 2.39, *p* < 0.001; **Figure 2B**) with no significant heterogeneity (*p* for the Cochrane Q test = 0.44; I^2^ = 0%). Sensitivity analyses excluding one study at a time did not materially change the results (HR: 1.95 to 2.28, *p* all < 0.05). Similar to the subgroup findings of OS, results of subgroup analysis also showed a stronger association between a low PNI and OS in OC patients with mean ages ≥ 65 years as compared to < 65 years (HR: 2.76 vs. 1.42, *p* for subgroup difference = 0.002; **Table 3**). Subgroup analyses also showed uniformly low heterogeneity across categories. No interaction was observed for the other variables in subgroup analyses. The results of univariate meta-regression analysis suggested a positive correlation between mean ages of the patients and HR for the association between PNI and PFS (coefficient = 0.055, *p* = 0.02; **Table 4**), which fully explained the variation of the results (adjsuted R^2^ = 100.0%). Other variables did not significantly modify the association between PNI and OS in patients with OC (*p* all > 0.05; **Table 4**).

### Publication bias

**Figure 3:**
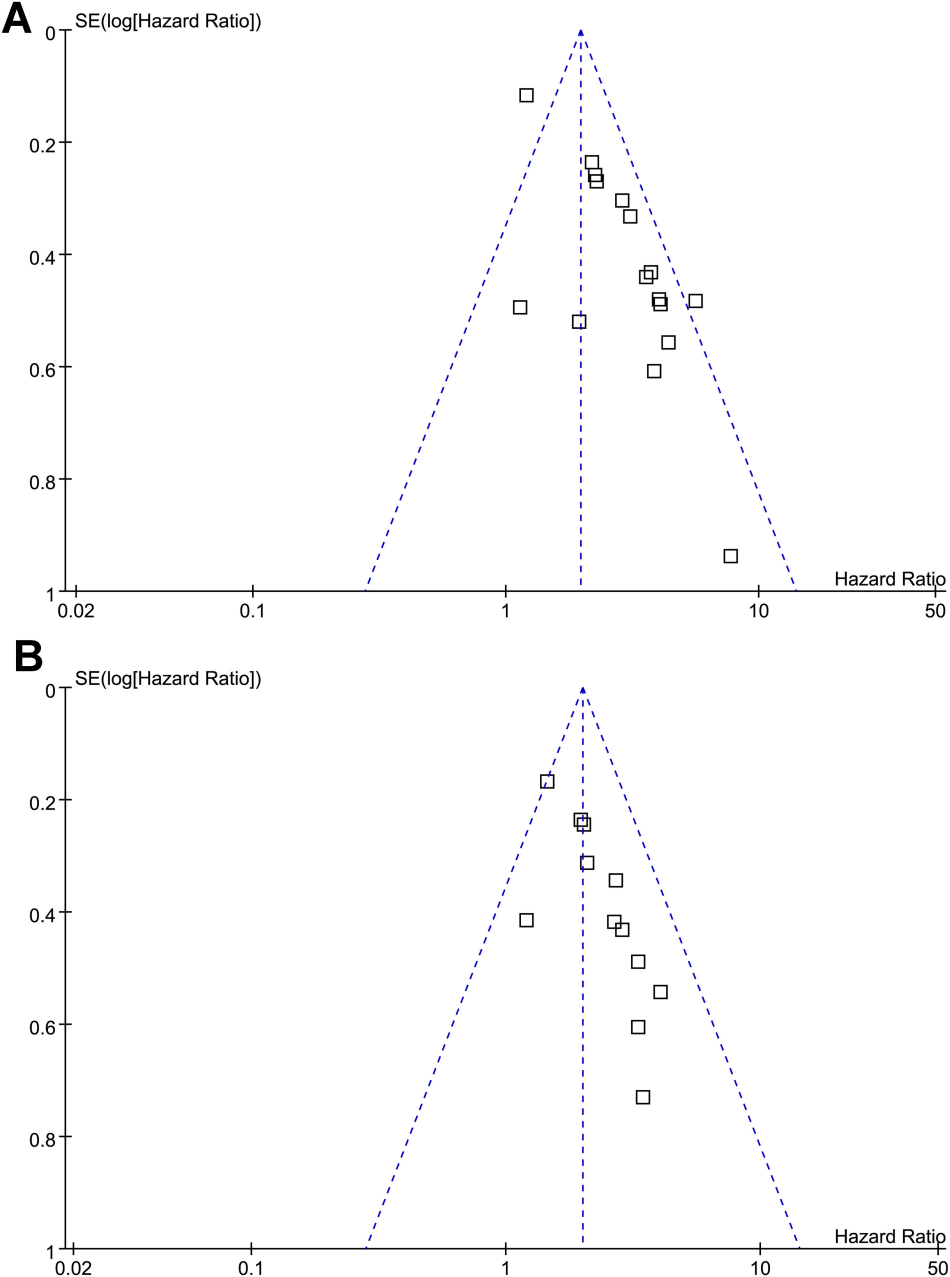
Funnel plots for estimating the potential publication biases underlying the meta-analyses of the association between a low PNI and survival outcomes of patients with OC; **(A)** funnel plots for the meta-analysis of the association between a low PNI and OS; and **(B)** funnel plots for the meta-analysis of the association between a low PNI and PFS; No significant publication bias was detected (Egger’s test: *p* = 0.41 for OS; *p* = 0.53 for PFS).

Funnel plots assessing the association between a low PNI and OS/PFS in patients with OC are presented in **Figures 3A, B**. The visual symmetry of the plots indicates a low likelihood of publication bias. In addition, Egger’s tests also confirmed the absence of significant publication bias (*p* = 0.39 and 0.33 for the outcome of OS and PFS, respectively).

## Discussion

This meta-analysis demonstrates that a low PNI is significantly associated with adverse survival outcomes in patients with OC. By quantitatively synthesizing data from 16 cohorts involving 3,520 patients, we found that patients with low PNI had markedly poorer OS and PFS, and this association was particularly strong among older individuals. These findings provide compelling evidence that PNI, a simple and inexpensive biomarker, holds considerable prognostic value in the management of OC. To our knowledge, only one previous meta-analysis has specifically examined the prognostic value of PNI in oral cancer, which included 10 studies with 3,130 patients (26). Our study substantially expands upon this work by incorporating 16 cohorts encompassing 3,520 patients and integrating newly published evidence up to June 2025. In contrast to the earlier analysis, we performed more comprehensive subgroup and meta-regression analyses to explore heterogeneity, applied random-effects models with robust variance estimation, and focused on both overall and progression-free survival outcomes. Importantly, our results newly identify patient age as a major source of heterogeneity, revealing a stronger prognostic impact of low PNI among older individuals. These methodological and analytical enhancements provide a more detailed and contemporary synthesis of the prognostic significance of PNI in oral cancer.

The biological rationale for the prognostic impact of PNI lies in the dual components of its calculation: serum albumin and peripheral lymphocyte count. Albumin, beyond its role as a nutritional marker, reflects systemic inflammatory status (32, 33). Hypoalbuminemia may indicate protein depletion, impaired hepatic synthesis, or increased catabolism associated with systemic inflammation (33, 34). Such alterations are known to reduce treatment tolerance, impair wound healing, and exacerbate the risk of postoperative complications (33, 34). Albumin also exerts antioxidant and anti-inflammatory effects, and its depletion contributes to oxidative stress and a prothrombotic state that may favor tumor progression and metastasis (35, 36). The second component, lymphocyte count, represents the host immune response (37). Lymphocytes, particularly cytotoxic T cells and natural killer cells, are central to antitumor immunity by mediating cytolytic activity and controlling micro-metastatic disease (37). Lymphopenia, therefore, reflects impaired immune surveillance and has been associated with poor responses to radiotherapy, chemotherapy, and immunotherapy (38). Recent evidence shows that high expression of ITGA5 and ITGA6 in head and neck cancers is associated with increased infiltration of immunosuppressive cells, reflecting an exhausted tumor immune microenvironment (39). Similarly, patients with low PNI often present systemic lymphopenia and immune suppression, suggesting that both molecular and host-level factors contribute to immune exhaustion in OC. In addition, recent evidence indicates that nicotine exposure can activate *CHRNA5* and the downstream mitogen-activated protein kinase kinase (MEK) and extracellular signal-regulated kinases (ERK) pathway, promoting tumor invasion and metastasis in head and neck cancers (40). As low PNI is often accompanied by heightened systemic inflammation involving similar signaling cascades, this suggests that inflammatory–nutritional imbalance may indirectly facilitate tumor progression through MEK/ERK pathway activation. These observations are consistent with the findings from a recent study, which elucidates that nutritional and inflammatory markers—including PNI—are closely linked with the tumor immune microenvironment and underlying genetic alterations that shape immune responses in OSCC (41). These findings support the concept that low PNI mirrors systemic malnutrition and impaired immune surveillance, thereby promoting tumor progression and unfavorable outcomes. However, while PNI reflects systemic nutritional and immune status, direct evidence linking it to the tumor immune microenvironment or immunotherapy response in OSCC remains limited. Further mechanistic studies are needed to clarify how nutritional–immune interactions reflected by PNI influence tumor-infiltrating lymphocytes and antitumor immunity.

On the other hand, recent clinical studies further support these mechanisms. Luo et al. reported that low PNI was predictive of postoperative complications, including infections and unplanned reoperations, in older OC patients undergoing free flap reconstruction (42). Wang et al. demonstrated that a preoperative PNI < 49.2 was independently associated with postoperative hypokalemia in OC patients (43), a complication linked to impaired recovery and increased perioperative morbidity. Other studies have similarly linked low PNI to higher risks of venous thromboembolism (44) and surgical site infection (45). These complications may delay adjuvant therapies and worsen long-term outcomes, reinforcing the plausibility of PNI as both a prognostic and predictive marker.

Moderate heterogeneity was observed for OS (I² = 64%), while heterogeneity for PFS was negligible (I² = 0%). Sequential sensitivity analyses demonstrated stable results, with pooled HRs ranging narrowly between 2.55 and 2.81 for OS and 1.95 to 2.28 for PFS, indicating no single study disproportionately influenced the results. Subgroup analyses reduced the I² for OS from 64% to below 25% in most stratified comparisons—particularly when analyses were restricted to older populations or high-quality studies—suggesting that age and methodological quality accounted for a substantial portion of variability. Meta-regression confirmed age as the main contributor (adjusted R² = 81.4%), whereas other factors, such as sex distribution, PNI cutoff values, follow-up durations, or study quality scores, showed no significant effect. Nevertheless, residual heterogeneity may still arise from differences in treatment modalities, baseline nutritional profiles, and timing or method of PNI assessment across studies, which warrants cautious interpretation of the pooled results. Nevertheless, the subgroup and meta-regression analyses provide important insights into the consistency and modifiers of PNI’s prognostic effect. The most notable finding was the significantly stronger association between low PNI and adverse outcomes among patients with a mean age ≥ 65 years. This is biologically plausible, as older individuals often have diminished physiological reserve, age-related sarcopenia, and immunosenescence, all of which magnify the consequences of poor nutritional and immune status (46). Moreover, malnutrition frequently coexists with frailty in this population, and studies suggest that the combination of malnutrition and frailty synergistically increases the risk of postoperative complications and mortality (47, 48). In contrast, subgroup analyses by sex, tumor stage, cutoff values, study design, analytic models, and study quality did not demonstrate significant differences, suggesting that the prognostic effect of PNI is broadly applicable across clinical subgroups.

This study has several strengths that enhance its credibility. First, the meta-analysis included a relatively large sample size drawn from 16 cohorts, ensuring adequate statistical power to detect associations. Second, the majority of included studies used multivariate analyses, which allowed adjustment for confounders such as age, sex, tumor stage, and treatment modality, thereby reducing bias. Finally, we performed comprehensive subgroup and meta-regression analyses, which identified age as a key source of heterogeneity, while ruling out other potential modifiers. Despite these strengths, several limitations should be considered when interpreting the results. Most included studies were retrospective in design, making them vulnerable to selection bias, incomplete follow-up, and unmeasured confounding (49). As patients with poorer nutritional or inflammatory profiles are more likely to have adverse outcomes, this could lead to a modest overestimation of the association between low PNI and poor survival. Conversely, incomplete adjustment for clinical and treatment-related factors in several cohorts might also cause underestimation of the true effect due to residual confounding. In addition, although we attempted to explore heterogeneity through subgroup and meta-regression analyses, individual patient data were not available, limiting our ability to evaluate the effects of comorbidities, detailed treatment modalities, or lifestyle factors. The cutoff values used to define low versus high PNI varied across studies (ranging from 36.4 to 52.4), and the lack of a standardized, universally accepted threshold remains a challenge for clinical implementation and comparability. Future research should aim to establish evidence-based cutoff values through large, prospective multicenter studies to enhance the reproducibility and clinical utility of PNI. Although subgroup analyses by disease stage (I–IV vs. advanced or recurrent cases) partly accounted for clinical variability, further stratification by treatment setting was not feasible because most studies did not report separate survival data for surgery, chemoradiotherapy, or immunotherapy. Differences in treatment modality and sequencing likely contributed to residual heterogeneity and may have influenced the strength of the observed associations. Future studies should report outcomes stratified by therapeutic approach to enable more refined analyses of PNI’s prognostic role across clinical contexts. Additionally, while most cohorts adjusted for major prognostic factors, residual confounding by unmeasured variables cannot be excluded. In addition, the findings of the meta-analysis should also be interpreted with caution given that all included studies were conducted in East Asian populations. Variations in baseline nutritional profiles, dietary habits, and body composition across regions may influence both absolute PNI values and their prognostic thresholds. Moreover, disparities in healthcare access, perioperative nutritional support, and oncologic treatment protocols could modify the relationship between PNI and outcomes. Therefore, external validation in non-Asian populations is warranted to confirm the generalizability of the observed associations and to establish region-specific reference values if necessary. Finally, as this is an observational synthesis, causality cannot be established, and it remains unclear whether interventions to improve PNI directly translate into improved survival. Future research should adopt prospective, multicenter cohort designs, employ standardized PNI definitions and measurement protocols, and pursue international validation to more accurately quantify the prognostic value of PNI and enhance its clinical applicability.

Clinically, PNI is an inexpensive, non-invasive biomarker derived from routine laboratory tests, making it practical for incorporation into everyday clinical practice (8). Compared with other inflammation-based indices such as the neutrophil-to-lymphocyte ratio, platelet-to-lymphocyte ratio, or Glasgow Prognostic Score, the PNI uniquely integrates nutritional (albumin) and immune (lymphocyte) components into a single metric. This dual representation may provide a more holistic reflection of host–tumor interactions and systemic resilience. Future head-to-head analyses are warranted to clarify if PNI performs comparably or even superiorly to these indices in OC. Moreover, identifying patients with low PNI at diagnosis or before treatment could enable risk stratification and tailored management strategies. For example, patients with low PNI may benefit from preoperative nutritional interventions, immunonutrition, or enhanced perioperative monitoring. In elderly patients, where the prognostic impact of PNI appears strongest, incorporating PNI into geriatric oncology assessments could improve decision-making regarding treatment intensity and supportive care. Moreover, given the evidence linking low PNI to postoperative complications, it may also serve as a useful tool in surgical risk assessment and optimization. Recent advances also highlight the emerging utility of PNI beyond prognostication, extending into perioperative risk modeling. A machine learning–based study in head and neck squamous cell carcinoma patients undergoing free flap reconstruction identified PNI, together with operation time and neutrophil-to-lymphocyte ratio, as key predictors of surgical site infection, outperforming traditional logistic regression models (50). This finding underscores the broader applicability of PNI as a marker of systemic resilience and supports its integration into predictive algorithms for optimizing surgical outcomes in oral and head-and-neck oncology. Moreover, our meta-analysis was based on a single pre-treatment PNI measurement, which may not fully capture fluctuations in nutritional and immune status throughout therapy. It remains unknown whether the dynamic changes in PNI during and after treatment may provide additional prognostic information. Future longitudinal studies should therefore evaluate the prognostic significance of dynamic PNI trajectories to better inform individualized patient management. Future research should also focus on several areas. Prospective multicenter studies are needed to validate the prognostic significance of PNI in diverse populations and to establish standardized cutoff values. Randomized controlled trials are warranted to determine whether interventions aimed at improving nutritional and immune status can translate into survival benefits for patients with low PNI. In addition, integrating PNI with other prognostic markers, such as frailty indices, systemic inflammation markers, or molecular tumor characteristics, may yield more comprehensive predictive models. Such models could help guide personalized treatment strategies, balancing oncological control with functional outcomes and quality of life.

## Conclusions

In conclusion, this meta-analysis provides up-to-date evidence that low PNI before treatment is associated with significantly poorer OS and PFS in patients with OC, with particularly pronounced effects observed in older individuals. These findings underscore the prognostic relevance of host nutritional and immune status and support the integration of PNI into routine prognostic assessment and clinical decision-making in OC. While further prospective validation is necessary, PNI represents a simple, cost-effective, and clinically meaningful biomarker that may improve risk stratification and ultimately contribute to better patient outcomes.

## Data Availability

The original contributions presented in the study are included in the article/**Supplementary Material**. Further inquiries can be directed to the corresponding authors.
