# Supplementary material for: Association between prognostic nutritional index and survival of patients with oral cancer: a meta-analysis
Source: Front Oncol. 2025 Nov 19;15:1698656. doi: 10.3389/fonc.2025.1698656 (PMC12672263; doi:10.3389/fonc.2025.1698656)
Supplement: Supplementary file 1 [file Table1.docx]

**PubMed (MEDLINE)**

(("prognostic nutritional index"[tiab] OR "prognostic nutrition index"[tiab] OR PNI[tiab]) AND ("Mouth Neoplasms"[Mesh] OR ("Carcinoma, Squamous Cell"[Mesh] AND ("Mouth"[Mesh] OR oral[tiab] OR "oral cavity"[tiab] OR mouth[tiab])) OR "Oral Squamous Cell Carcinoma"[tiab] OR "oral cancer"[tiab] OR "oral cavity cancer"[tiab] OR "mouth neoplasm*"[tiab] OR "oropharyngeal cancer"[tiab] OR "oral-pharyngeal cancer"[tiab] OR OSCC[tiab]) AND ("Mortality"[Mesh] OR "Prognosis"[Mesh] OR "Survival"[Mesh] OR "Neoplasm Recurrence, Local"[Mesh] OR "Disease-Free Survival"[Mesh] OR "Progression-Free Survival"[Mesh] OR "Disease Progression"[Mesh] OR mortality[tiab] OR prognosis[tiab] OR survival[tiab] OR death*[tiab] OR recurren*[tiab] OR "overall survival"[tiab] OR "disease-free survival"[tiab] OR "progression-free survival"[tiab] OR "relapse-free survival"[tiab] OR progression[tiab])) AND ("1900/01/01"[Date - Publication]: "2025/06/08"[Date - Publication]) AND English[lang]

**Embase (Ovid syntax)**

(('prognostic nutritional index' OR 'prognostic nutrition index' OR pni).ti,ab,kw. AND ('mouth neoplasm'/exp OR 'mouth cancer'/exp OR 'oral squamous cell carcinoma'/exp OR 'oropharyngeal cancer'/exp OR 'oral squamous cell carcinoma'.ti,ab,kw. OR 'oral cancer'.ti,ab,kw. OR 'oral cavity cancer'.ti,ab,kw. OR 'mouth neoplasm*'.ti,ab,kw. OR 'oropharyngeal cancer'.ti,ab,kw. OR 'oral-pharyngeal cancer'.ti,ab,kw. OR oscc.ti,ab,kw.) AND ('mortality'/exp OR 'prognosis'/exp OR 'survival'/exp OR 'disease free survival'/exp OR 'progression free survival'/exp OR 'neoplasm recurrence'/exp OR 'disease progression'/exp OR mortality.ti,ab,kw. OR prognosis.ti,ab,kw. OR survival.ti,ab,kw. OR death*.ti,ab,kw. OR recurren*.ti,ab,kw. OR 'overall survival'.ti,ab,kw. OR 'disease-free survival'.ti,ab,kw. OR 'progression-free survival'.ti,ab,kw. OR 'relapse-free survival'.ti,ab,kw. OR progression.ti,ab,kw.)) AND [english]/lim AND [1900-2025]/py

**Web of Science**

TS=(("prognostic nutritional index" OR "prognostic nutrition index" OR PNI) AND ("oral squamous cell carcinoma" OR "oral cancer" OR "oral cavity cancer" OR "mouth neoplasm*" OR "oropharyngeal cancer" OR "oral-pharyngeal cancer" OR OSCC) AND (mortality OR prognosis OR survival OR death* OR recurrence OR "overall survival" OR "disease-free survival" OR "progression-free survival" OR "relapse-free survival" OR progression)) AND LANGUAGE: (English) AND Timespan: 1900-2025
